# HOTSPOT: An ad hoc teamwork platform for mixed human-robot teams

**DOI:** 10.1371/journal.pone.0305705

**Published:** 2024-06-28

**Authors:** João G. Ribeiro, Luis Müller Henriques, Sérgio Colcher, Julio Cesar Duarte, Francisco S. Melo, Ruy Luiz Milidiú, Alberto Sardinha

**Affiliations:** 1 INESC-ID, Instituto Superior Técnico, Universidade de Lisboa, Lisboa, Portugal; 2 Departamento de Informática, Pontifícia Universidade Católica do Rio de Janeiro, Rio de Janeiro, Brasil; 3 Seção de Ensino de Engenharia de Computação, Instituto Militar de Engenharia, Rio de Janeiro, Brasil; CINVESTAV IPN: Centro de Investigacion y de Estudios Avanzados del Instituto Politecnico Nacional, MEXICO

## Abstract

Ad hoc teamwork is a research topic in multi-agent systems whereby an agent (the “ad hoc agent”) must successfully collaborate with a set of unknown agents (the “teammates”) without any prior coordination or communication protocol. However, research in ad hoc teamwork is predominantly focused on agent-only teams, but not on agent-human teams, which we believe is an exciting research avenue and has enormous application potential in human-robot teams. This paper will tap into this potential by proposing HOTSPOT, the first framework for ad hoc teamwork in human-robot teams. Our framework comprises two main modules, addressing the two key challenges in the interaction between a robot acting as the ad hoc agent and human teammates. First, a *decision-theoretic module* that is responsible for all task-related decision-making (task identification, teammate identification, and planning). Second, a *communication module* that uses natural language processing to parse all communication between the robot and the human. To evaluate our framework, we use a task where a mobile robot and a human cooperatively collect objects in an open space, illustrating the main features of our framework in a real-world task.

## Introduction

Recent decades have witnessed a significant shift in the use of robots. While robotic platforms still find extensive use in industry, advances in hardware and software have enabled the development of various robotic platforms for everyday use. For instance, robots are being used in healthcare [1], assisted living [2], entertainment [3], and even for mundane tasks such as cleaning [4]. Moreover, as the use of robots broadens beyond industrial applications, the ability of these platforms to naturally interact with users who have no technical expertise becomes a mandatory requirement. Thus, it is not surprising that the area of human-robot interaction has seen impressive growth in the last decades.

In this paper, we are particularly interested in *collaborative* human-robot interaction. This topic is not novel, and a significant body of literature has investigated human-robot collaboration from many different perspectives [5, 6]. However, very few works focus on the problem of *ad hoc teamwork* involving a human and a robot.

Ad hoc teamwork was proposed originally in the multi-agent systems community [7] and addresses the problem of an agent (henceforth called the “ad hoc agent”) that must successfully cooperate with a group of unknown “teammates”—i.e., other agents about which the ad hoc agent has little or no information. This group of agents must now act as a *team*, even if they have no prior cooperation or coordination mechanisms. The role of the ad hoc agent is to understand or infer *what* is the task that the other agents are performing, *who* among the other agents is doing what, towards the completion of the task, and then decide *how* to contribute. These three challenges were identified in Melo and Sardinha [8] as fundamental sub-tasks of the ad hoc teamwork problem, dubbed *task identification*, *teammate identification* and *planning*.

So far, research in ad hoc teamwork has focused primarily on agent-agent interaction scenarios [9], and rests on strong assumptions regarding what the ad hoc agent is able to perceive regarding the environment, the teammates, or the task to be addressed. Dealing with teams of humans and robots brings forth several critical challenges that current research on ad hoc teamwork has not considered yet. For example:

- Robots, as embodied agents, have to deal with perceptual and actuation challenges that virtual agents seldom consider. In particular, the robot’s perception of its state is often imperfect, and its actuation is prone to failure;
- The teammate—being a human—does not behave according to a well-defined model (for example, it is not necessarily optimal or rational);
- Decision-making must be conducted at run-time.

These challenges are common in human-robot interaction scenarios but rarely considered in the ad hoc teamwork literature (if at all). Additionally, humans can communicate through natural language, and such communication channels can be rich and informative if the robot can take advantage of them. However, dealing with natural language is another challenge for robot developers, although the potential applicability of ad hoc teamwork in everyday tasks is enormous and mostly untapped.

This paper’s main contribution is a framework for ad hoc teamwork between a human and a robot. The ad hoc robot knows there is a human performing an unknown task and that there is no pre-coordination between them, for instance, with the human telling the robot any information regarding the correct task. Instead, the robot must rely on prior knowledge to identify what the human is doing and assist the human effectively. In this work, we model the robot’s prior knowledge base as a library of possible tasks that the robot may have performed in the past. We leave beyond the scope of the work, scenarios where the task being performed by the human was never performed by the robot. Our framework, which we dub HOTSPOT (HOTSPOT stands for “Human-robOt TeamS without PrecoOrdinaTion”.), instantiates the problem of ad hoc teamwork in human-robot teams as follows. A human and a robot co-exist in a shared environment and must perform a collaborative task that requires them to coordinate their actions. The human knows the task, but the robot (the ad hoc agent in our setting) does not. From observations of the human, the robot must infer the task (among a set of possible tasks), understand how the human user is performing it, and adapt its decision process toward completing the task.

In our scenario, we consider tasks in an open space (i.e., a lab), where the human and the robot must move around. This requirement poses additional challenges to the robot due to the semi-unstructured interaction, where the robot will not know the location of the human user most of the time. To address this ad hoc teamwork problem, we propose a *decision-theoretic approach* that extends the one proposed by Ribeiro et al. [10] to account for the perceptual limitations of the ad hoc agent (the robot). At the same time, to take advantage of the fact that human users can communicate using natural language, we endow the robot with the ability to *communicate* with them. In particular, the robot can communicate through spoken utterances, querying the user about the task’s current state, interpreting the human’s response using natural language processing, and considering the inherent uncertainty in that interpretation process.

We evaluate the HOTSPOT framework in a real-world scenario involving the interaction between a human and a robot in an open space, evaluating the performance of each module individually and of the whole framework. To summarize, the contributions of this work are three-fold.

- We contribute a *decision-theoretic model for ad hoc teamwork with limited perception*. We describe the decision problem faced by the ad hoc agent (the robot) as a *partially observable Markov decision problem* and use a standard heuristic solution to efficiently compute an adequate policy for the robot. Our results show that our approach can infer and complete the task in a near-optimal number of steps while still using partial and imperfect information.
- We demonstrate that via a *natural language processing model* our framework allows the robot to understand the utterances issued by the human, use that information to locate itself in the environment, and express itself in an easy way that the human can understand. Our results show that these models achieved an accuracy of about 80% in every task performed.
- We contribute an empirical validation of our approach in a real-world ad hoc teamwork scenario involving a human and a mobile robot.

## Related work

This section frames our contribution in the context of existing research both on *ad hoc teamwork* and *natural language processing* since these are the two areas of research most relevant to our present work.

Regarding ad hoc teamwork, the problem was originally proposed in the pioneering work of Stone et al. [7] and has spanned a significant volume of research [8, 10–12]. Following Melo and Sardinha [8], we can break down the ad hoc teamwork problem into three main sub-problems: *task identification*, *teammate identification*, and *planning*.

Early research into ad hoc teamwork focused on the *planning* step. For example, Stone and Kraus [13] proposed one of the first planning algorithms for ad hoc teamwork, by formulating the problem as a cooperative *k*-armed bandit with known teammates. Similarly, Stone et al. [14] and Agmon and Stone [15] looked at ad hoc teamwork as a problem of “leading” known teammates to perform actions that yield optimal joint performance.

Genter et al. [16] focused on the problem of *teammate identification*, proposing a role-based approach allowing an agent to identify a role, within *m* roles, that yields optimal performance for the team. Chakraborty and Stone [17] presented a learning algorithm and a theoretical analysis regarding optimal cooperation in the presence of unknown teammates.

Nikolaidis et al. [18] proposed a framework, for learning human user models in collaborative tasks with robots through joint-action demonstrations, that assumes a limited number of dominant strategies, capturing most demonstrated sequences and modeling human preference as a partially observable variable in a mixed-observability Markov decision problem. These demonstrated sequences are then clustered into human types, and a reward function representative of each kind is learned using inverse reinforcement learning. Experiments conducted with a human subject indicate stronger agreement that the robot anticipated actions with the proposed framework compared to a manual annotation process, improving team efficiency, and increasing the responsiveness to human actions compared to hand-coded policies.

Barrett et al. [9] introduced the PLASTIC framework to address ad hoc teamwork when facing both unknown tasks and teammates, using a reinforcement learning approach. To this day, the PLASTIC algorithms remain among the state-of-the-art in ad hoc teamwork, addressing both *task and teammate identification*.

On the other hand, Reddy et al. [19] discussed how suboptimal behavior can result from a disconnect between an individual’s mental model of the world and its actual dynamics, indicating that what seems suboptimal in reality may align closely with a user’s internal beliefs. It emphasizes humans’ reliance on intuitive theories to navigate complex environments, which can lead to behaviors deviating from optimal outcomes due to simplified internal models. It also proposes an algorithm for inferring users’ intents based on their internal dynamics model, which is learned from observed behaviors. The method’s effectiveness is presented through simulations and a user study, suggesting potential applications in shared autonomy and inverse reinforcement learning, however, limitations exist regarding the size of parameterizations for internal dynamics, suggesting avenues for future research to explore broader applications and scientific inquiries, such as understanding children’s intuitive theories and enhancing brain-computer interfaces.

Bobu et al. [20] presented a novel approach, named LESS (Limiting Errors due to Similar Selections), that generalizes the Luce axiom to trajectory spaces by considering trajectory similarity’s influence on probability. Through experiments and simulations, the authors showed that LESS improves the prediction of human decision-making, better explaining human behavior, while also enhancing inference accuracy and robustness compared to a simple Boltzmann model. Nevertheless, limitations such as reliance on pre-specified robot features for similarity selection and the need for further experiments to clarify outcomes are pointed out. Despite these, this highlights the importance of developing alternatives to Boltzmann rationality for continuous robotics domains.

Li et al. [21] discussed the challenge of an agent collaborating with an unknown human, addressing the issues of predicting human behavior and choosing actions towards a common goal. Considering multiple human types with diverse models, it employs self-play to identify optimal partnerships between agent types in a strategic game scenario called Team Space Fortress. The proposed method treats human behavior as a factored Markov decision problem, while also introducing a new adaptive agent framework based on cross-entropy similarity measures and a pre-trained policy library. Their evaluation confirms the effectiveness of the adaptive agents, although limitations exist, suggesting future work could improve the representativeness of this library for better estimation of human policies.

Melo and Sardinha [8] addressed the three sub-problems of ad hoc teamwork by proposing two distinct approaches. Specifically, one approach is based on sequential prediction [22] and the other one on decision-theoretic planning [23]. The two approaches consider one-shot problems and, as such, are not suited for sequential problems. Along the same lines, Ribeiro et al. [10] extended the previous work to address sequential tasks under uncertainty.

Most previous works, however, consider only agent-agent interaction in that they disregard critical difficulties found when an embodied agent (such as a robot) must interact with human teammates. A human-robot interaction setting must consider the limitations of using a robotic platform (such as unreliable and limited perception and unreliable actuation) and the interaction with a human user.

Nevertheless, some recent works take important steps towards bringing ad hoc teamwork closer to human-robot interaction scenarios. For example, in terms of ad hoc teamwork involving robots, Genter et al. [24] investigated the use of ad hoc algorithms in the RoboCup World Championship, in the context of the Drop-in Player Competition [25].

Fern et al. [26] addressed the problem of *assistance* which, although not formulated as an ad hoc teamwork problem, shares many of its challenges. Specifically, in assistance problems, an agent (the “assistant”) aims to assist a teammate in solving a given sequential task under uncertainty. In a closely related work, Ribeiro et al. [10] already considered ad hoc teamwork involving a human teammate. However, both works consider perfect observability and do not leverage the communicating capabilities of human users.

The two modules in our proposed architecture extend, on one hand, the decision-making process of Ribeiro et al. [10] to accommodate partial observability. On the other hand, the communication module enables our robot to leverage the communication capabilities of the human user toward the completion of the joint task.

Regarding Natural Language Processing (NLP), this is a field of computer science that enables machines to understand and process human communication [27] by transforming unstructured data, like audio or text, into structured data, which are more suitable for machines. It can also work in the opposite direction by generating communication for humans to understand easily.

Many works address the use of NLP in interaction with robots. Scheutz et al. [28] presented the challenges of designing mechanisms that allow robots to develop human dialogues in interactions between humans and robots. In addition to building a small survey of the area, its main objective is to help build better, more flexible robotic architectures that can enable more natural language dialogues between humans and robots. Furthermore, the authors briefly propose DIARC, a Distributed, Integrated, Affective, Reflective, and Cognitive architecture that allows robotic systems to conduct human dialogues without providing much detail about the techniques involved in the process.

On the other hand, Kilicaslan and Tuna [29] explored the use of NLP resources to improve human-robot interaction through the use of ontologies that represent the grammatical and lexical structures of the language. Implementations have been generated for English and Turkish languages to allow the robot to express the spatial motions of observed objects. Through the use of these ontologies, a representation of the robot’s observation can be described by a tuple <Location, Source, Goal, Path>, where Location refers to the position of the observed object, Source represents where the object moved from, Goal refers to the target position of the object, and Path represents the trajectory that the object has made. A prototype was built using the ROS framework, where a camera captures object movements in front of the robot to describe the movement in natural language.

Briggs et al. [30] used pragmatic and dialogue-based mechanisms to understand typical human directives and create suitable responses. Specifically, utterances are used to represent the speech act classification, as well as the speaker, robot, and semantics analysis. Then, rules associate the utterances with a tuple containing the set of inferred beliefs based on the intended meaning of the utterance. For instance, a question inquiring whether some assertive is true or false. Finally, a dialogue-based mechanism handles and generates the responses based on expectations generated by the utterances. Experiments were then conducted to show the viability of the proposed methodology in identifying indirect speech acts and coverage of the utterance forms.

Li et al. [31] used NLP to infer human-given commands for robots, by using keyword extraction, visual object recognition, and similarity computation. Its main intent is to use visual semantic information to allow a robot to deduce task intents, avoiding simple keywords that map predefined tasks explicitly. The proposed method uses rule matching and conditional random fields to analyze and extract information from the processed sentences.

Despite several works that use NLP in robots, none of them are tailored to the ad hoc teamwork scenario. Our work thus presents a novel contribution to the scientific literature, namely an architecture that combines decision-making and NLP for human-robot collaboration within ad hoc teamwork settings.

## The HOTSPOT framework

This section presents the HOTSPOT framework for ad hoc teamwork involving humans and robots, which is the key contribution of this paper. When performing ad hoc teamwork between humans and robots, three main challenges arise.

First, the environment is not fully observable, as the robot has to rely on sensors to obtain its perceptions. In fully observable environments, agents are assumed to have access to the current state of the environment, without any errors or imperfect information. With a properly modeled fully observable state (i.e., one that contains all information relevant to the task at hand) given to an agent, optimal planners can be used to compute optimal actions. In a partially observable environment, a state is still assumed to exist, but not observable to the agent (or in this case, the robot). In real-world environments, it is not feasible to, in real time, compute the current state of the environment to allow the robot to plan for optimal action. By using the robot’s sensors, however, one may compute a partial observation of such state, and use internal models within the robot to infer the most-likely states and act accordingly. In the HOTSPOT framework, we assume the robot to have only access to partial observations, acquired via its sensors.

Secondly, when interacting with humans as teammates, it is not feasible to assume they will explicitly communicate their actions in real time, as robots would possibly communicate between them if they all shared the same communication protocol (even though in the setting of ad hoc teamwork communication between agents is also assumed to not be always possible due to the fact that the robot knows nothing about the team’s members). For this reason, the robot has to rely on other information to infer what the human teammate is doing. In the HOTSPOT framework, we mitigate this issue with two solutions: (i) we assume the human may sometimes communicate with the robot (even though this may never happen at all), and the robot has to be fully prepared to understand the human’s voice and (ii) the actions of the human influence the state of the environment, which in turn influences the partial observations made by the robot. We propose, respectively, a module capable of parsing the human’s voice and converting it into a possible action and an approach to infer the most likely action given the partial observations of the environment.

Furthermore, it is also not feasible to assume the behavior of the human user will always be optimal and efficient (as humans may stop what they are doing without warning or even act inefficiently). This challenge requires robots to be robust to sub-optimal behavior. In the HOTSPOT framework, we assume that even though human teammates know the task being performed, they may suddenly not act or act sub-optimally, evaluating how we are able to mitigate this issue by using teammate models of the humans.

**Fig 1:**
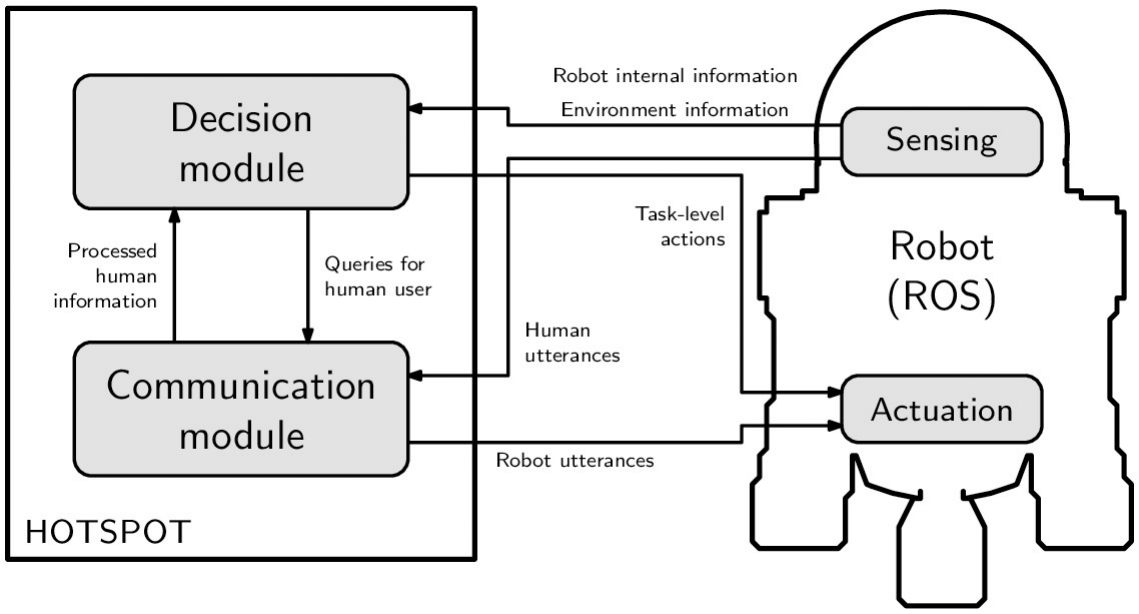
HOTSPOT diagram. Diagram depicting the interaction between the different modules in HOTSPOT.

Fig 1 presents the two main modules in the HOTSPOT architecture, namely:

- *A decision module* that is responsible for ad hoc teamwork decisions with the human. This module receives as input the robot and environment information from the sensors and the relevant information from the human speech (i.e., the information processed by the communication module). The decision module then uses such information to reconstruct/estimate the current state of the environment and the human-robot team. Finally, the robot uses the state information to estimate the current task (*task identification*), to identify how the human is executing such task (*teammate identification*), and to act accordingly (*planning*).
- *A communication module* that is responsible for the communication with the human user. It receives queries from the decision module and translates them into spoken utterances that the robot must execute (verbalize and animate). It is also responsible for processing human utterances, as perceived by the sensors, providing the decision module with their relevant information.

In the remainder of this section, we describe both modules in greater detail.

### Decision module

**Fig 2:**
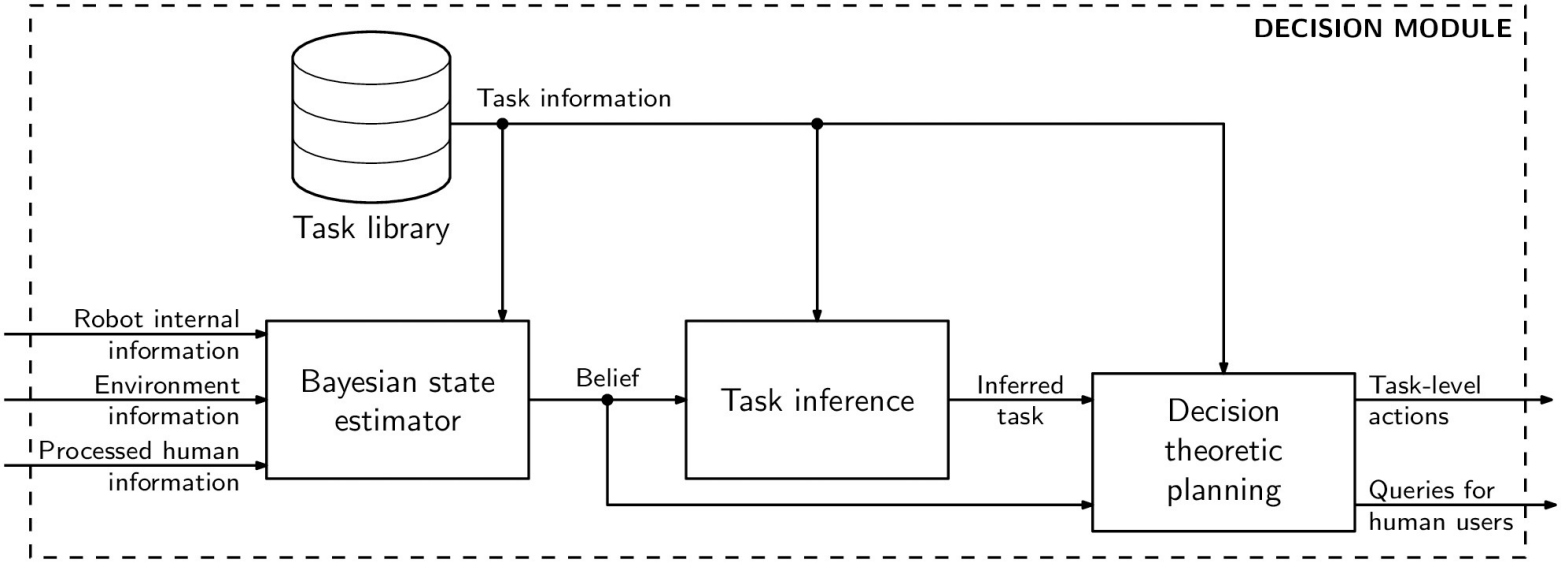
HOTSPOT decision module diagram. Diagram depicting the decision module of HOTSPOT.

The decision module is depicted in Fig 2. The module processes the information coming from the sensors and communication module to estimate the *state* of the current task. Specifically, HOTSPOT maintains a distribution over possible states—a *belief*—which is updated from the perceived information using a standard Bayesian update.

The robot then uses information about possible tasks (stored in a *task library*) to infer the current task by checking which tasks in the library are most likely to yield the perceived information from the environment and the teammate. Finally, using the belief and task information, the robot plans the actions to complete the task. It also determines which (if any) communication actions it should perform toward the human.

In the continuation, we formalize each of these processes in detail.

#### Task description

We build on the works of Ribeiro et al. [10] and Melo and Sardinha [8] to formalize the possible tasks of the human-robot team using a decision-theoretic framework that accommodates the inherent uncertainty in scenarios involving robots in a principled manner.

We represent each possible task in the agent’s library as a *Multiagent Markov Decision Problem* (MMDP) [32], consisting of a tuple $M_{m}=\left(X,A_{0},A_{-0},P_{m},r_{m},\gamma \right)$, where X is the set of all possible states, $A_{0}$ is the set of actions available to the robot, *A*_−0_ is the set of actions available to the teammate (the human user), **P**_*m*_ describes the *dynamics* of the task *m*, *r*_*m*_ is a reward function, describing the *goal* of task *m*, and *γ* is a discount factor.

The states encode all task-relevant information, i.e., all information that, at each time step, the agents (the robot and the human) require to decide the next action. We write *X*_*t*_ to denote the state at time-step *t*, and *A*_0,*t*_ and *A*_−0,*t*_ to denote the actions of the robot and the human user at time-step *t*, respectively. The state evolves according to the transition probabilities in **P**_*m*_, i.e., if *T* denotes the (unknown) current task,

$$\begin{array}{c} P_{m}\left(x^{\prime }|x,a_{0},a_{-0}\right)=P[X_{t+1}=x^{\prime }|X_{t}=x,A_{0,t}=a_{0},A_{-0,t}=a_{-0},T=m], \end{array}$$ (1)

with $x,x^{\prime }\in X$, $a_{0}\in A$, and $a_{-0}\in A_{-0}$. The transition probabilities describe the effect that the actions of the robot and the human user have on the *state*, given that the current task is *m*. Similarly, the reward function *r*_*m*_ encodes the goal of the human-robot team: the value *r*_*m*_(*x*, *a*_0_, *a*_−0_) measures the *instant utility* of the robot executing action *a*_0_ and the human executing action *a*_−0_ in the state *x*, when the task is *m*.

Together, the human and the robot want to select their actions to maximize the total sum of rewards which, if *T* = *m*, can be written as

$$\begin{array}{c} J=E[\sum_{t=0}^{\infty }\gamma^{t}r_{m}\left(X_{t},A_{0,t},A_{-0,t}\right)], \end{array}$$ (2)

where *γ* ∈ [0, 1) is a discount factor assigning greater value to rewards arriving earlier than those arriving later. Solving an MMDP thus consists of computing two individual *policies* for the two agents, *π*_0_ and *π*_−0_, each prescribing an action for each possible state so that the prescribed actions jointly solve the MMDP—i.e., maximize the value in (2). Solving an MMDP can be done using standard dynamic programming techniques such as value or policy iteration [33].

In our setting, we consider that there is a set of *M* possible tasks, each described as an MMDP $M_{m}=(X,A_{0},A_{-0},P_{m},r_{m},$ *γ*), where all tasks share the state and action spaces but may have different dynamics and goals. Furthermore, we assume that the robot does not know beforehand which task is being performed—henceforth referred to as the *target task* *T*—but the human user does know. Task identification thus consists of inferring the target task from the information that the robot can observe during the interaction.

#### Bayesian state estimator

During the interaction, the robot can observe the information available through its sensors and information provided by the communication module regarding the human spoken utterances. We denote by *Z*_*t*_ the information observed by the agent—which we assume takes values in a finite set of possible observations, Z. We also denote by **O** the *observation probabilities*, which essentially provide a probabilistic description of the sensing process of the robot. In particular,

$$\begin{array}{c} O\left(z|x,a_{0}\right)=P[Z_{t+1}=z|X_{t+1}=x,A_{0,t}=a_{0}], \end{array}$$

with z∈Z and x∈X. The observation probabilities describe how likely it is for the robot to observe *z* in state *x*, given that the last action of the robot was *a*_0_.

Let *b*_*t*_(*x*) denote the probability that, at time step *t*, the state is x∈X, given the history of observations and actions of the robot up to that time step (henceforth *H*_*t*_). Let us further assume that, at time step *t*, the robot performs the action *a*_0_, and the human user performs the action *a*_−0_. As a consequence, the environment will transition to state *X*_*t*+1_ and the robot will observe *Z*_*t*+1_ = *z*. Then, if the target task is *m*, we can update our belief *b*_*t*_ using a standard Bayesian update to have

$$\begin{array}{c} \begin{array}{cc} b_{t+1}\left(x\right) & =P[X_{t+1}=x|H_{t+1}]\\ & =\frac{1}{\rho }\sum_{x^{\prime }\in X}P_{m}\left(x|x^{\prime },a_{0},a_{-0}\right)O\left(z|x^{\prime },a_{-0}\right)b_{t}\left(x^{\prime }\right), \end{array} \end{array}$$

where *ρ* is a normalization constant.

There are two difficulties with using this update: first, we do not know which is the action of the human teammate, *a*_−0_; and second, we do not know which is the target task, *m*.

To address the first difficulty, and since we assume that the teammate knows the target task, we consider that—if the target task is *m*—the action of the teammate can be *any* optimal action for the task *m*. Then, if we average out the action selection of our human teammate, we get the (task-dependent) transition probabilities

$$\begin{array}{c} {\bar{P}}_{m}\left(x^{\prime }|x,a_{0}\right)=\frac{1}{\left|A_{-0}^{*}\left(x\right)\right|}\sum_{a_{-0}\in A_{-0}^{*}\left(x\right)}P_{m}\left(x^{\prime }|x,a_{0},a_{-0}\right), \end{array}$$

where $A_{-0}^{*}\left(x\right)$ denotes the set of optimal teammate actions in state *x*. This yields a task-dependent belief update

$$\begin{array}{c} \begin{array}{cc} b_{m,t+1}\left(x\right) & =P[X_{t+1}=x|H_{t+1},T=m]\\ & =\frac{1}{\rho }\sum_{x^{\prime }\in X}{\bar{P}}_{m}\left(x|x^{\prime },a_{0}\right)O\left(z|x^{\prime },a_{-0}\right)b_{m,t}\left(x^{\prime }\right), \end{array} \end{array}$$ (3)

where *ρ* is, again, a normalization constant.

Regarding the second difficulty, since the robot does not know beforehand the target task, it maintains a distribution *p*_*t*_ over the set of possible tasks. In other words, we write *p*_*t*_(*m*) to denote the probability that the target task is *m* given the history of observations and actions of the robot up to time step *t*. Then, given the distribution *p*_*t*_, we can write the “average” belief at time step *t* as

$$\begin{array}{c} b_{t}\left(x\right)=\sum_{m=1}^{M}p_{t}\left(m\right)b_{m,t}\left(x\right). \end{array}$$ (4)

#### Task inference

We now describe how to maintain the distribution *p*_*t*_, used to perform Bayesian state estimation. Much like with the state estimation, we adopt a Bayesian framework. Let *T* denote the unknown target task. Then, if the agent observed *z* at time step *t* + 1 after executing *a*_0_ at time step *t*,

$$\begin{array}{cc} p_{t+1}\left(m\right) & =P[T=m|H_{t+1}]\\ & =\frac{1}{\rho }P[Z_{t+1}=z|A_{0,t}=a_{0},T=m,H_{t}]P[A_{0,t}=a_{0}|H_{t}]p_{t}\left(m\right), \end{array}$$

where, once again, *ρ* is the necessary normalization constant. We used the fact that the action selected by the agent at each moment depends only on the history of observations and not on the target task *T*. Then,

$$\begin{array}{c} P[Z_{t+1}=z|A_{0,t}=a_{0},T=m,H_{t}]=\sum_{x,x^{\prime }\in X}O\left(z|x^{\prime },a_{0}\right)P_{m}\left(x^{\prime }|x,a_{0}\right)b_{m,t}\left(x\right). \end{array}$$

#### Decision-theoretic planning

To decide what action to take, and given the uncertainty in the robot’s perception of its state, we adopt as the planning approach a well-established information-gathering heuristic [34]. In particular, at each time step *t*, the robot selects its actions to balance *information gathering* and *task completion*.

Information gathering consists of selecting actions that decrease the uncertainty in the state estimation, *b*_*t*_. Task completion actions are selected to solve the target task, *T*.

To this purpose, we compute the *normalized entropy* of *b*_*t*_, given by

$$\begin{array}{c} \bar{H}\left(b_{t}\right)=-\frac{1}{\text{log}\;|X|}\sum_{x\in X}b_{t}\left(x\right)\;\text{log}\;b_{t}\left(x\right). \end{array}$$

The normalized entropy measures the uncertainty in the agent’s belief. Let *b*_max_(*z*, *a*_0_) denote the robot’s belief upon observing *z* after executing *a*_0_ in task *m* from a belief with maximum entropy, i.e.,

$$\begin{array}{c} b_{m,\text{max}}\left(z,a_{0}\right)=\frac{1}{\rho }\sum_{x\in X}{\bar{P}}_{m}\left(x^{\prime }|x,a_{0}\right)O\left(z|x^{\prime },a_{0}\right)\frac{1}{\left|X\right|}. \end{array}$$

We define the *information gain* associated with (*z*, *a*_0_) as

$$\begin{array}{c} \Delta H_{m}\left(z,a_{0}\right)=1-\bar{H}\left(b_{m,\text{max}}\left(z,a_{0}\right)\right). \end{array}$$

We also define the *reward gain* associated with (*z*, *a*_0_) as the maximum reward that the robot can get upon observing *z* after executing *a*_0_ in task *m* from a belief with maximum entropy, i.e.,

$$\begin{array}{c} \Delta R_{m}\left(z,a_{0}\right)=\underset{a_{0}^{\prime }\in A_{0}}{\text{max}}\;\sum_{x,x^{\prime }\in X}{\bar{P}}_{m}\left(x^{\prime }|x,a_{0}\right)O\left(z|x^{\prime },a_{0}\right)\frac{{\bar{r}}_{m}\left(x^{\prime },a_{0}\right)}{\left|X\right|}, \end{array}$$

with

$$\begin{array}{c} {\bar{r}}_{m}\left(x,a_{0}\right)=\frac{1}{\left|A_{-0}^{*}\left(x\right)\right|}\sum_{a_{-0}\in A_{-0}^{*}\left(x\right)}r_{m}\left(x,a_{0},a_{-0}\right). \end{array}$$

Following Melo and Ribeiro [34], we define an *information gathering reward function* as

$$\begin{array}{c} r_{m,\text{info}}\left(x,a\right)=\sum_{z\in Z}P[Z_{t+1}=z|X_{t}=x,A_{0,t}=a_{0},T=m]\Delta H_{m}\left(z,a_{0}\right)\Delta R_{m}\left(z,a_{0}\right). \end{array}$$

Then, for each task *m*, we can now define two standard MDPs, $\left(X,A_{0},{\bar{P}}_{m},r_{m},\gamma \right)$ and $\left(X,A_{0},{\bar{P}}_{m},r_{m,\text{info}},\gamma \right)$, which can be solved to yield two optimal *Q*-functions [33], $Q_{m}^{*}$ and $Q_{m,\text{info}}^{*}$.

Finally, the action selected at each step *t* is given by

$$\begin{array}{c} a_{0,t}=\text{argmax}\;\sum_{m=1}^{M}p_{t}\left(m\right)\sum_{x\in X}b_{t}\left(x\right)[\left(1-\bar{H}\left(b_{t}\right)\right)Q_{m}^{*}\left(x,a\right)+\bar{H}\left(b_{t}\right))Q_{m,\text{info}}^{*}\left(x,a\right)]. \end{array}$$

When the uncertainty in *b*_*t*_ is high (close to 1), the robot selects an information-gathering action, i.e., an action that maximizes $Q_{m,\text{info}}^{*}$; when the entropy is low (close to 0), the robot selects a task competing action, i.e., an action that maximizes $Q_{m}^{*}\left(x,a\right)$.

### Communication module

**Fig 3:**
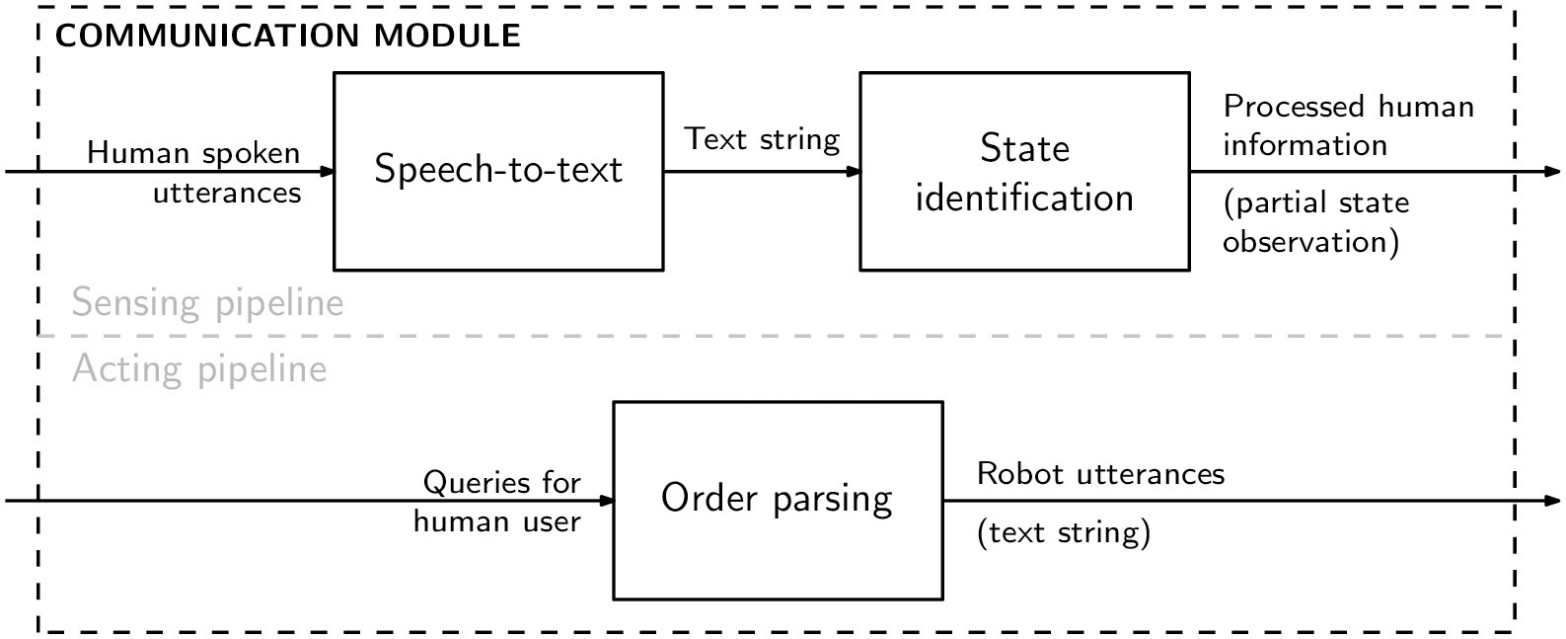
HOTSPOT communication module diagram. The communication module, comprising both a *sensing* pipeline that converts speech to state information, and a *actuation* pipeline, converting communicating actions to text to be then spoken by the robot.

Fig 3 depicts the communication module, which plays two roles in the overall HOTSPOT architecture. First, it plays a *sensing role*, transforming the human speech (captured through a microphone) into state information that is then used by the decision module. Second, it also plays an *acting role*, converting the communication actions received from the decision module into utterances that the robot then speaks to the human user. Each role corresponds to a well-defined pipeline, as depicted in Fig 3.

Concerning the sensing pipeline, the communication between the robot and the human user occurs through speech, captured by a microphone, and transformed into audio data, usually in the form of a .wav file containing the recorded human spoken utterances. Next, the audio data is transcribed by a speech-to-text block into a text format that better represents the audio in the language being spoken. Subsequently, in the state identification block, the transcribed text is passed into an NLP processor to extract semantic information, which is then translated into a partial state description.

The description of the state is then used as one of the several inputs to the decision module, which returns action(s) based on the behavior explained in the Decision Module section. These actions are mapped both into task-level and communication actions. The latter actions are passed to the communication module once again (i.e., the acting pipeline).

On the other hand, the acting pipeline is responsible for converting the communication actions into a text string, which is then sent to the robot. Specifically, this is done by the order parsing module, which provides the utterances to the robot, thus closing the cycle of communication between HOTSPOT and the robotic platform.

### Interaction with the robot

The interaction between HOTSPOT and the robot relies on the *robot operating system* (ROS) [35], which is responsible for sensor handling (namely, processing all sensor readings), robot control, and communication. In other words, ROS supervises all sensing and actuation of the robot, and it is through a ROS interface that HOTSPOT interacts with the robot.

In particular, ROS collects all sensor data, arriving both from the robot sensors—such as odometry sensors used in dead-reckoning, lasers, contact sensors, etc.—and environment sensors—such as microphones, cameras, and other sensors that may exist in the environment. The speech data is sent to the communication module, while the remaining sensor data is processed and sent to the decision module.

ROS is also responsible for the actuation of the robot. In particular, it receives the task-level actions (such as moving) from the decision module and the text strings (corresponding to the utterances that the robot should speak) from the communication module and performs these on the robot.

## Experimental setup

To evaluate the effectiveness of our approach, we created a controlled environment where a live robot interacts with a human teammate. In this environment, the robot aims to assist the human in cleaning up a room, with the interaction being restricted by a set of rules from the Toxic Waste Domain.

### The toxic waste domain

The Toxic Waste domain has a two-agent team composed of a human cleaner and a robot container. The team is in a building with several rooms and has to clean three rooms with toxic or radioactive waste. A specific task from this domain lays out the rooms in a topological map, where the nodes represent the rooms, and the edges represent the doors that connect them. In addition, some rooms may contain toxic material on the ground. Hence, in each time step, both the human and the robot may choose to move from one room (node) to another or stay in the same node (i.e., no-op).

Whenever it finds itself in a node containing toxic waste, the human can pick it up from the ground or release it (if already holding it). After picking up the toxic waste, the human must remain standing still on his current node and wait for the robot to get close to disposing of the toxic material into the robot’s container. The robot also has an additional action to query the human by location (which the human may or may not respond to). Finally, in each time step, the robot receives its current location as an observation, inferred by the dead reckoning module.

**Fig 4:**
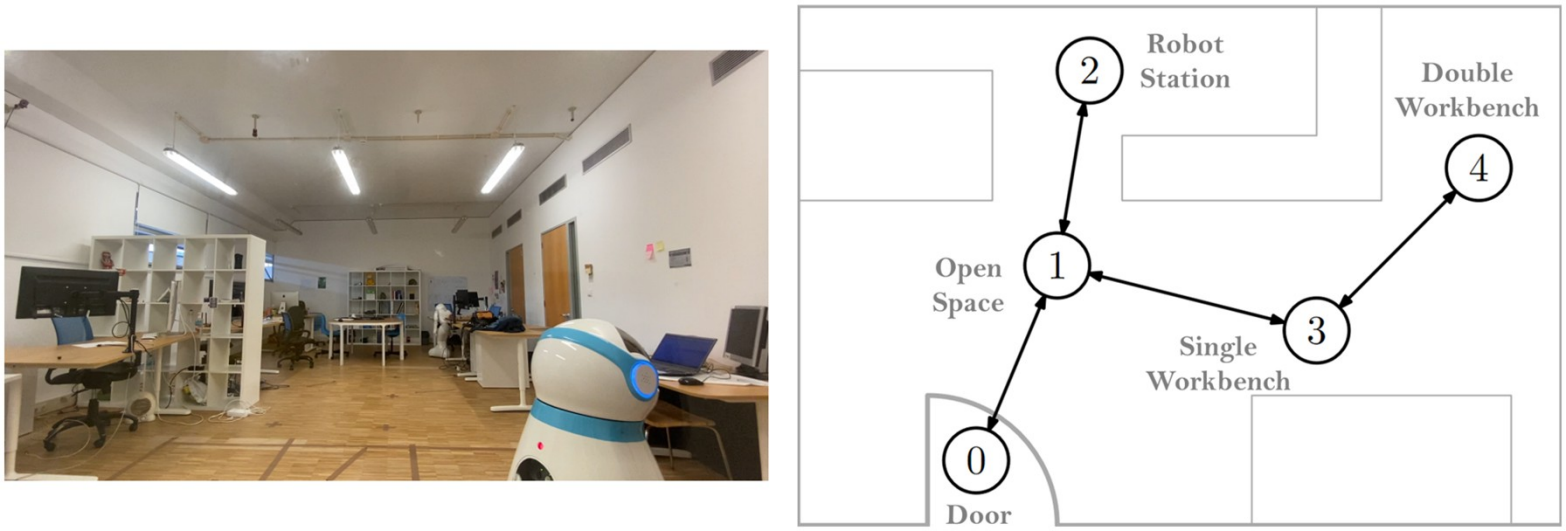
Lab room setup. Lab room used to simulate the Toxic Waste domain (left) and respective layout (right). Each area is represented as a node in a topological map.

**Fig 5:**
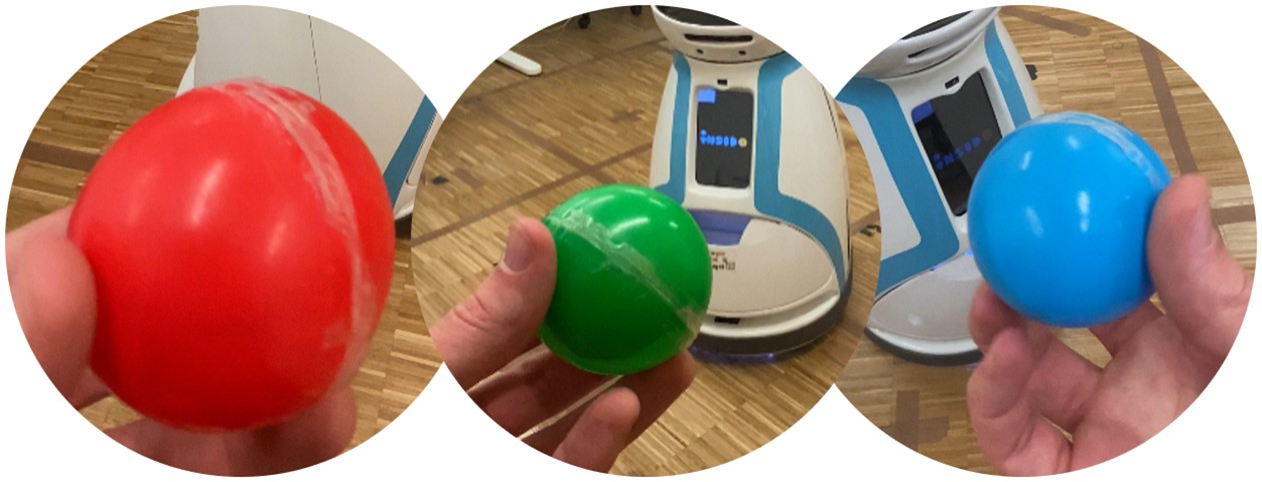
Toxic waste materials. The three balls representing the toxic waste materials.

**Fig 6:**
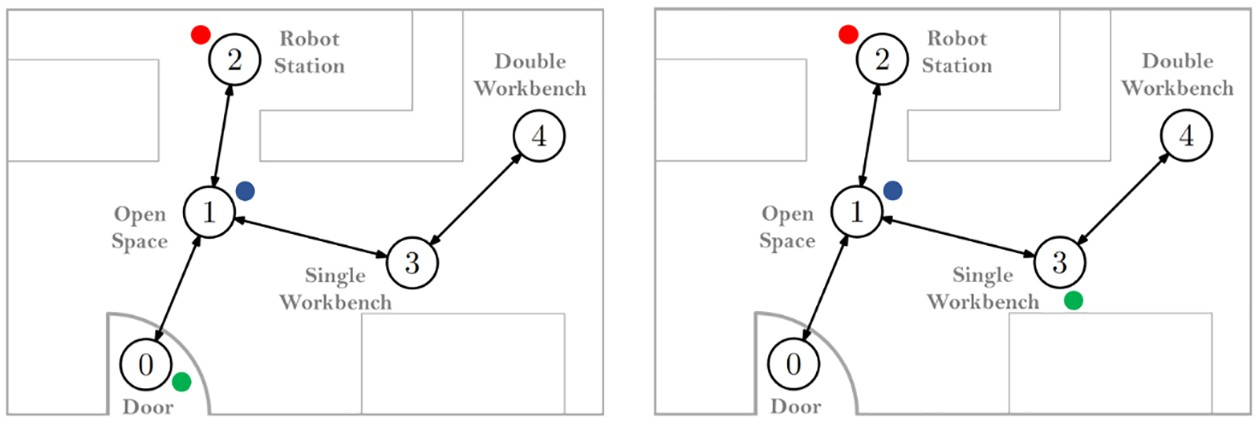
Task configurations. The two task configurations (i.e., locations of the three balls representing the toxic waste materials).

Fig 4 shows the Toxic Waste domain that we created within our laboratory. Precisely, we recreate two tasks by dividing our laboratory room into five distinct areas, representing separate rooms: 0—door, 1—open space, 2—robot station, 3—single workbench, and 4—double workbench. To represent the toxic waste that humans can collect, we use three colored balls placed in three different nodes, as depicted in Fig 5. The location of the three balls models each task; that is, there are two possible tasks, each with the three balls placed in three respective areas, as shown in Fig 6.

To represent the human cleaner, we rely on people from a small focus group who have been told the goal in advance and know how to act according to the domain’s rules, namely: i) they may only make one move at a time, ii) they may reply to the robot’s questions, and iii) they may only move from one area to the another if they are connected. We rely on Astro (Fig 4—left) to be the robot container. It is a robot from our laboratory capable of moving around the room and possessing a front recipient equipped with an RFID sensor to detect when a ball is placed inside the recipient. For each person in the focus group, we randomly chose a task from the two possible tasks, with the human starting in the area of their preference and the robot always starting at the door.

### The decision module

We instantiate the decision module as a Python 3 ROS node running on a laptop (connected via wifi to a ROS master node running on the robot). The implementation of the module requires only a library of possible tasks, which are then used by our Bayesian state estimator, task inference, and decision-making algorithm.

To model the tasks in the Toxic Waste domain in our framework, we must specify each corresponding MMDP, as well as the observation space and probabilities, describing the sensing process of the robot. Each MMDP is a tuple $\left(X,A_{0},A_{-0},P_{m},r_{m},\gamma \right)$ with distinct transition probabilities and reward functions. Together with the specification of the observation space and observation probabilities for the robot, they provide all the necessary information required by the decision-making module.

**Fig 7:**
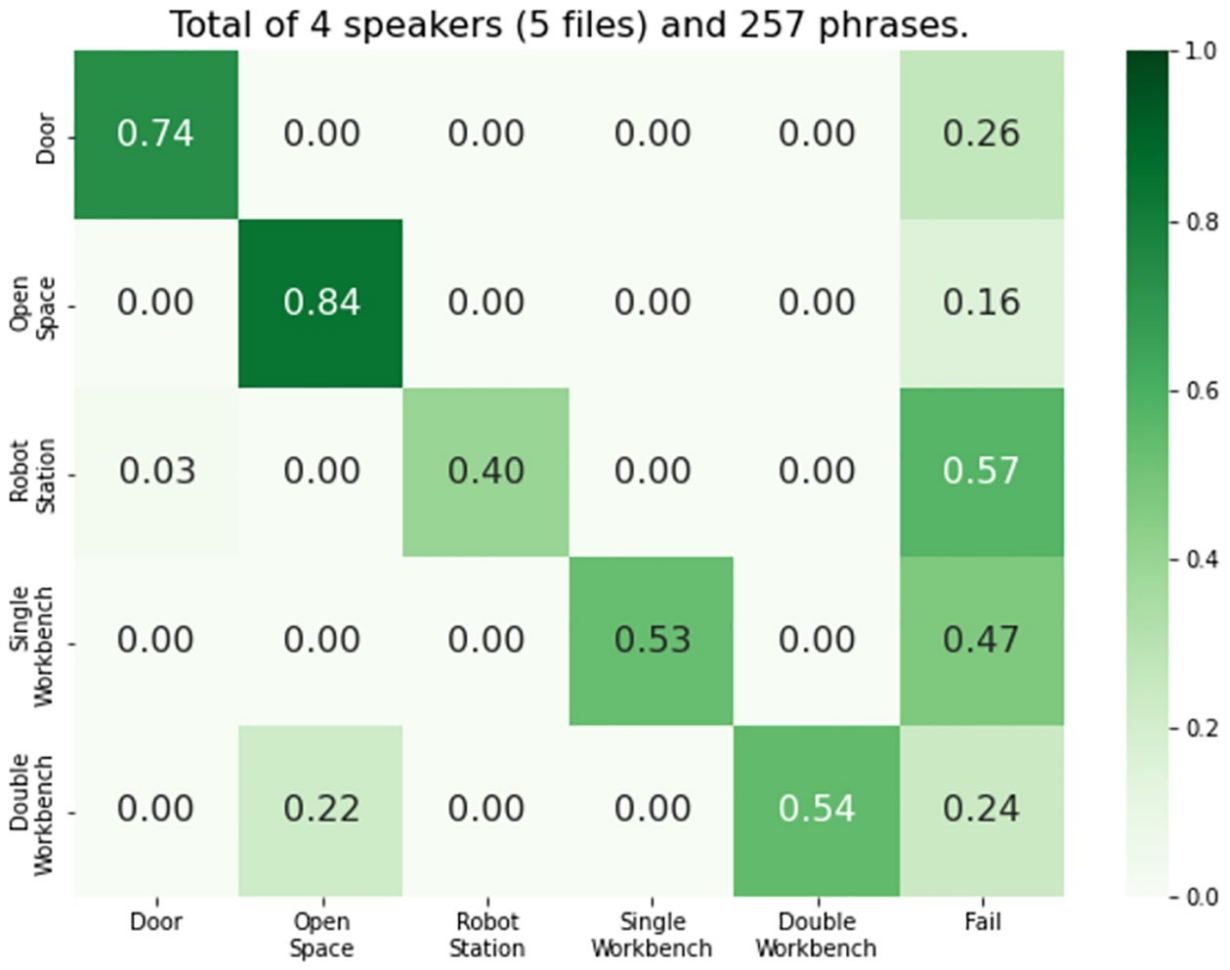
Confusion matrix. Communication module’s pipeline confusion matrix.

In our experiments,

- the state space X contains information regarding both agents’ nodes and the status of the three toxic materials (i.e., on the ground, picked up, or disposed of). In particular, a state x∈X is a tuple (*n*_*r*_, *n*_*h*_, *w*_1_, *w*_2_, *w*_3_), where *n*_*r*_ and *n*_*h*_ represent the node of the robot and human, respectively, and *w*_*i*_ represents the status of the toxic waste material *i*.
- the action space for the robot, $A_{0}$, has five possible actions available: *move the lowest-index node*, *move the second lowest-index node*, *move the third-index node*, *stand still*, and *ask the human for his location*. The human action space, $A_{-0}$, has similar move actions and, additionally, a *pick waste* and *drop waste* actions.
- the transition probabilities **P**_*m*_ describe how the robot and human move as a consequence of their movement actions, and the status of the waste material as a consequence of the actions of the human user.
- the reward functions *r*_*m*_ assign a penalty of −1 for each toxic waste on the ground, −2 for each toxic waste on the human’s hands, and 0 for each toxic waste material disposed of.
- we use a discount *γ* = 0.95.
- the observations describe what the robot can observe regarding the state of the environment and the human user. Specifically, each observation z∈Z corresponds to a tuple $\left({\hat{n}}_{r},{\hat{n}}_{h},rfid\right)$, where ${\hat{n}}_{r}$ represents the robot’s node (determined via dead reckoning) and ${\hat{n}}_{h}$ represents the human’s node (determined from speech). *rfid* is a boolean flag indicating that the container detected the collection/disposal of toxic waste.
- as for the observation probabilities **O**, regarding the location of the robot and RFID sensor, we empirically assessed that the error in these measurements was negligible. As for the position of the human (perceived from human speech), we ran a preliminary study where, for each possible human node, we script out several phrases from a small focus group (i.e., 4 different speakers reading 257 different phrases). We then build a confusion matrix (Fig 7) using Python’s machine learning library scikit-learn, which tells, for each true node, the probability of identifying every other node or even failing to identify any node. Finally, to handle live errors, we smooth the probabilities when loading the model using the confusion matrix to ensure that no entry has an absolute zero.

#### The human teammate

When it comes to modeling human behavior in the toxic waste domain, we model the human teammate as an agent that knows the task being performed and fully observes the environment, unlike the ad hoc robot which has to identify the task and is only able to partially observe the environment.

In each time step *t*, the human teammate has a probability *ϵ* of not selecting the optimal action $a_{-0}^{*}$, computed from the optimal policy $\pi_{-0}^{*}\left(x_{t}\right)$. This parameter represents the probability of the human not moving, simulating distractions such as looking at the phone, talking with someone, or moving to a non-optimal node by mistake. Furthermore, and although the option of replying to the robot’s action ‘locate human’ is not part of the human’s MMDP action space $A_{-0}$, in the POMDP’s observation probabilities **O** for the robot action *locate human*, we consider an additional probability of the human not successfully replying to the robot. This takes into consideration situations where, for any particular reason, the human doesn’t reply at all or situations where the communication module fails to correctly interpret the human’s reply.

### Metrics

Given that our approach has several independent modules, we evaluate the system with the following metrics: i) the number of steps it takes to solve a task, ii) TEBOPA’s number of steps to identify the correct task, iii) entropy in TEBOPA’s belief over tasks, iv) communication module’s speech recognition accuracy, v) communication module’s named entity recognition accuracy, vi) communication module’s node location recognition accuracy and vii) robot’s dead-reckoning accuracy in identifying the correct node. We now break down each individual metric.

#### Metric 1—The number of steps it takes to solve a task

Our first and main metric used was the number of steps to solve a task. This metric is used to assess the performance of the team, and the fewer the number of steps it takes the team to solve a task, the better. In our real-world scenario, a third-party human assistant, observing the experiment, registers the number of steps until the task is considered complete, i.e., when all three balls have been placed within the robot’s compartment.

#### Metric 2—TEBOPA’s number of steps to identify the correct task

Our approach, TEBOPA, keeps a belief over the possible tasks, as it interacts with the environment. Each entry in this belief vector measures the likelihood of the respective task. As the robot interacts with the environment, our algorithm TEBOPA, performs a Bayesian update to these probabilities using the observations made in each step. We log, in each step, the task with the highest belief, which represents the task our approach ‘guesses’ as being the correct one. When, in a given time step T, the likelihood of the correct task becomes the highest in the belief vector, and stays so until the end of the interaction, we consider it took our approach T steps to identify the correct task.

#### Metric 3—Entropy in TEBOPA’s belief over tasks

As detailed in our description of our second metric, our approach, TEBOPA, keeps a belief over the possible tasks, as it interacts with the environment. One metric we can therefore use to measure the ‘uncertainty’ of our agent, is the entropy of the belief vector. The lower the entropy, the more ‘certain’ our agent is of the task being performed. We compute the entropy for a trial after the trial ends, relying on the belief vectors logged by our decision module.

#### Metric 4—Communication module’s speech recognition accuracy

Our fourth metric measures the accuracy of the first component of our communication module, the speech recognizer. The speech recognizer takes as input the audio of a speaker and outputs the respective text. Unlike the prior metrics, this metric is computed prior to the experiments, when setting up the confusion matrix for the model of the environment that needs to take into account the probability of failing to interpret the human’s utterances. Each speaker is told to read a list of phrases, and then the resulting text output matched against the original phrase. Some nuances such as the difference in capital letters of punctuation were ignored if they did not grammatically change the phrase.

#### Metric 5—Communication module’s named entity recognition accuracy

Our fifth metric measures the accuracy of the named entity recognition component of our communication module. Like the previous metric, it was also measured prior to the experiments, as required by the model of the environment to take into account the probability of failure when modeling the POMDP. Taking as input the text output of the speech recognition component, the named entity recognition component outputs a location entity, such as ‘door’ or ‘table’. Associated with each human phrase, the correct entity is labeled. If the output of the named entity recognition component matched the location entity for the respective phrase, we would consider the entity correctly identified.

#### Metric 6—Communication module’s node location recognition accuracy

Another communication module’s metric is the node location recognition accuracy. The node location recognition component of our communication module takes as input the entity from the named entity recognition component, which it then uses to predict the correct node. A node label is given to each phrase and used to check if the output is correct. Like our previous two metrics, this metric was also assessed prior to the trials, when computing the confusion matrix and failure probabilities for the models used by the agent.

#### Metric 7—Robot’s dead-reckoning accuracy in identifying the correct node

Our seventh and final metric measures the number of correct node identifications made by the robot’s dead-reckoning system. Each time the robot moves from one node to another, it computes the new position by adding the required offset into its current one. Afterward, the robot moves into the new position and assumes to be there, a process known as dead-reckoning. Each point from the robot’s internal referential is then mapped into the area of the nodes from the Toxic Waste domain, and, as such, the robot is able to ‘guess’ its own node. A third-party human observer then registers the nodes the robot navigates throughout the trial, while the decision module also registers the ‘guessed’ nodes. These two lists are then used to compute the accuracy of the dead-reckoning sensors.

### Participants and procedure

In this section, we describe how our participants were recruited and briefed, and how our experimental procedure was conducted.

#### Selection of the focus group

We recruited a total of seven members from our laboratory, *pro bono*, for the empirical evaluation of the HOTSPOT framework. The minimum age was 23 years and the maximum was 42. When contacting each participant, no details regarding the trials were discussed, only a date scheduled. On the date and at the time of the trial, participants were told by an experiment moderator what the task consisted of—having to pick up the three balls from the floor and deposit them on the robot only when the robot would share the node with them. They were told they were unable to move if they had picked up any balls and the dynamics were turn-based. No two participants from the focus group observed each other’s trials.

#### Monitoring of the trials

Every trial was monitored by one of the authors (João G. Ribeiro) in the role of a moderator. The moderator starts by briefing the participant on what the task consisted of, and the aforementioned rules. The moderator then attached the microphone to the participant’s clothes and told the participant it was free to answer the robot when asked questions. Afterward, the moderator would ask the participant to select a starting location, and chose, at random, both a task and a starting location for the robot which did not match the location of the human. The trial would then start. Finally, the moderator manually registered both the node of the robot and the node of the participant in each time step. After the task was completed, the moderator would also register the total number of steps it took the human and the robot to complete the task.

#### Selection of the task and starting positions

All tasks were chosen at random by the moderator before knowing the starting position of the robot and participants. Before placing the balls on the task’s location nodes, the participants would be allowed to choose their own location and move to their starting position. After the participants reached their starting position, the moderator manually controlled the robot to its randomly chosen starting position, ensuring it did not contain any balls. The trial would then start.

#### Choice of performing more than a single trial

We allowed participants who wished to run additional trials to do so, after completing their first trial. In such cases, these participants would perform a different task, excluding the performed one from being selected at random by the moderator. The same procedure would then be repeated. Out of the 7 participants recruited, only 2 decided each to run an additional trial.

#### Additional baseline approaches

Finally, to assess the introduced difficulties associated with live robotic experiments, we evaluate three agents in a simulated environment: (i) our own approach, TEBOPA, to compare how the results from the simulated environment differ from the ones obtained in the real-world experiments; (ii) an agent following an optimal policy, able to fully observe the state of the environment, used as a reference for best possible performance; (iii) an ablation of TEBOPA which knows the task beforehand, used as a reference for optimal behavior under partial observability and (iv) an agent following a random policy, used as a reference for worst possible performance.

## Results

**Fig 8:**
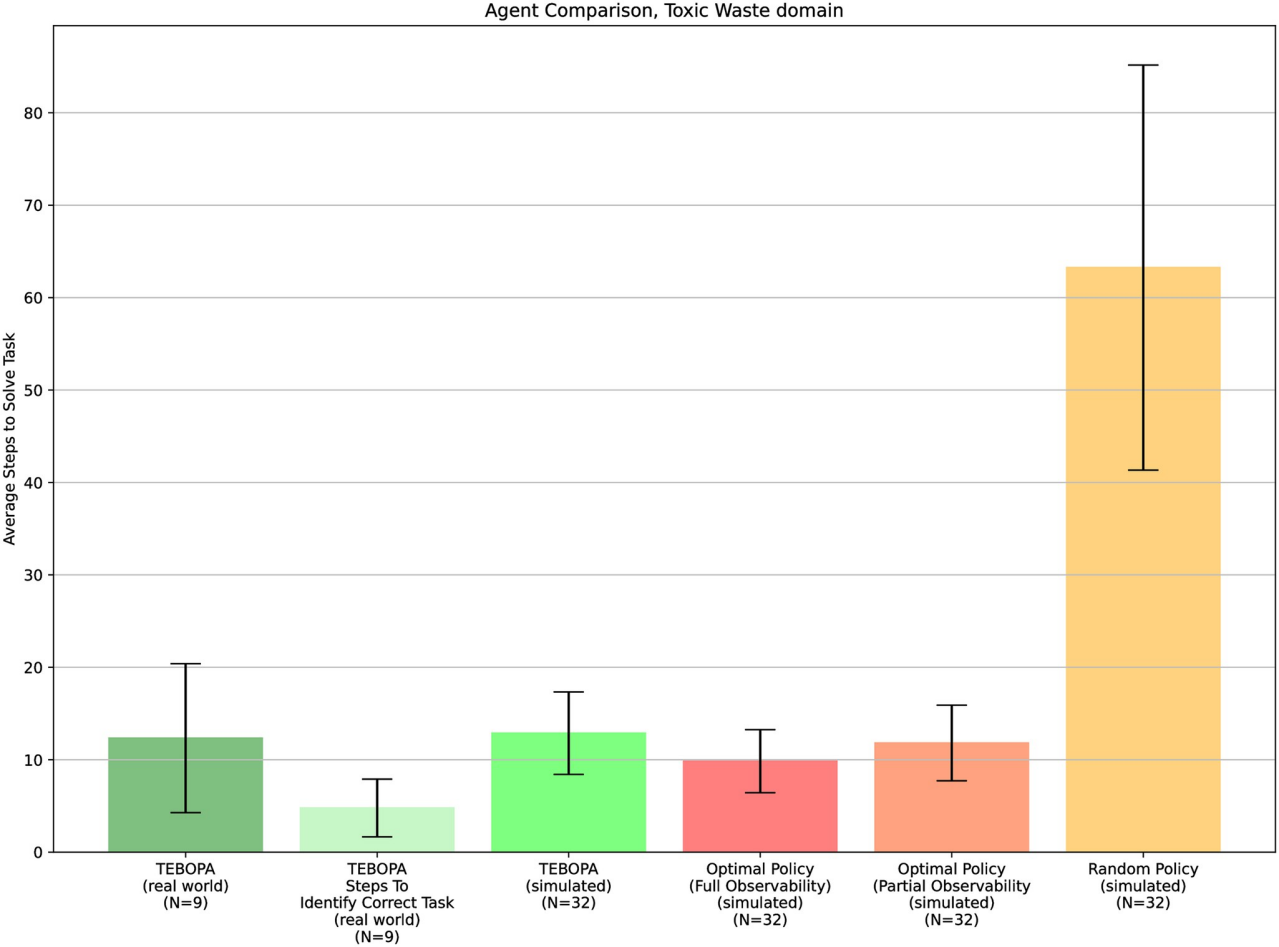
Average number of steps required to solve and identify the target task was analyzed across different scenarios, considering the use of the communication module. All simulated trials started from the same initial states as those in the live trials. Error bars correspond to a confidence interval with the confidence of 95% (*α* = 0.05%), calculated over all trials, encompassing 9 real-world trials and 32 trials in the simulated environment).

Fig 8 shows the average number of steps our approach took to complete the task assigned to the team. It also plots the number of steps required for our approach to identify the correct task from two possible tasks. We also present the number of steps it takes for four baseline agents to run in a simulated environment, namely (i) an agent following an optimal policy, representing the best possible performance (ii) an agent following a random policy, representing the worst possible performance, (iii) our approach in the simulated environment, to compare with the results obtained in the real world, further validating both our simulated environment and human model, and (iv) an ablation of our approach which knows the task beforehand and therefore represents an optimal agent under partial observability.

From the results presented in Fig 8, we observe that our approach always completed the target task in a near-optimal number of steps and quickly identified the target task using only partial observations (without observing the human’s actions). As expected, the agent following the optimal policy solved the tasks in fewer steps than the random policy under partial observability (*p* = 0.0000018, *α* = 0.05), our approach, TEBOPA, (*p* = 0.00757, *α* = 0.05) and the random policy (*p* = 0.00000000007, *α* = 0.05). Our approach, TEBOPA, was able to not only solve tasks in fewer steps than the random policy (*p* = 0.0000000001887, *α* = 0.05), but was also able to solve tasks in the same average number of steps as the optimal policy under partial observability (*p* > *α* = 0.05). We can also observe that, on average, our approach can identify the correct task quicker than an optimal policy can solve it (*p* = 0.0028, *α* = 0.05).

Furthermore, our results for the multiple participants, which were allowed to pick their starting nodes, do not hint at any benefits or disadvantages of starting at any specific node. Finally, we can observe that the results obtained for our approach in the simulated environment matched the results obtained by our approach in the real world, with no statistically significant difference (*p* > *α* = 0.05). This demonstrates the validity of not only our simulated environment but also our model of the human teammate.

**Fig 9:**
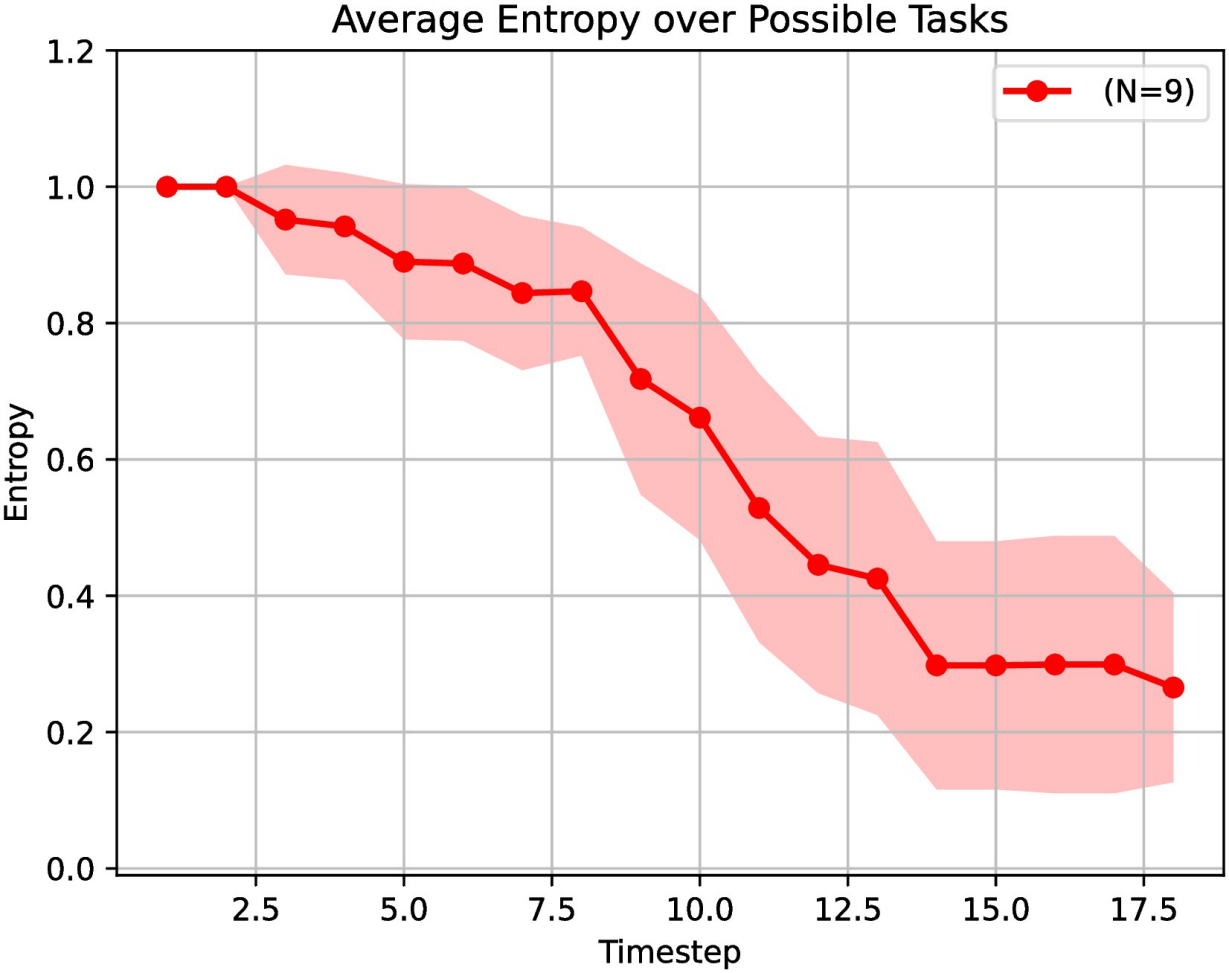
Beliefs entropy. Entropy of the beliefs at each time step, averaged over all nine trials;

We can look deeper into the task identification by plotting the average entropy of the beliefs at each time step, as shown in Fig 9.

We can first observe in Fig 9 that the average entropy decreases with each passing time step. This is expected because the agent has more information to infer the correct task as the agent interacts with the environment. The second observation is that the average entropy does not reach 0.0, although it has dropped from 1.0 to almost 0.20. This result shows that our approach may end a trial without being 100% sure what the correct task is. However, this also indicates that our approach effectively solves the most likely task. Additionally, the fact that the average entropy is not 0.0 means that it may recover from task changes.

**Fig 10:**
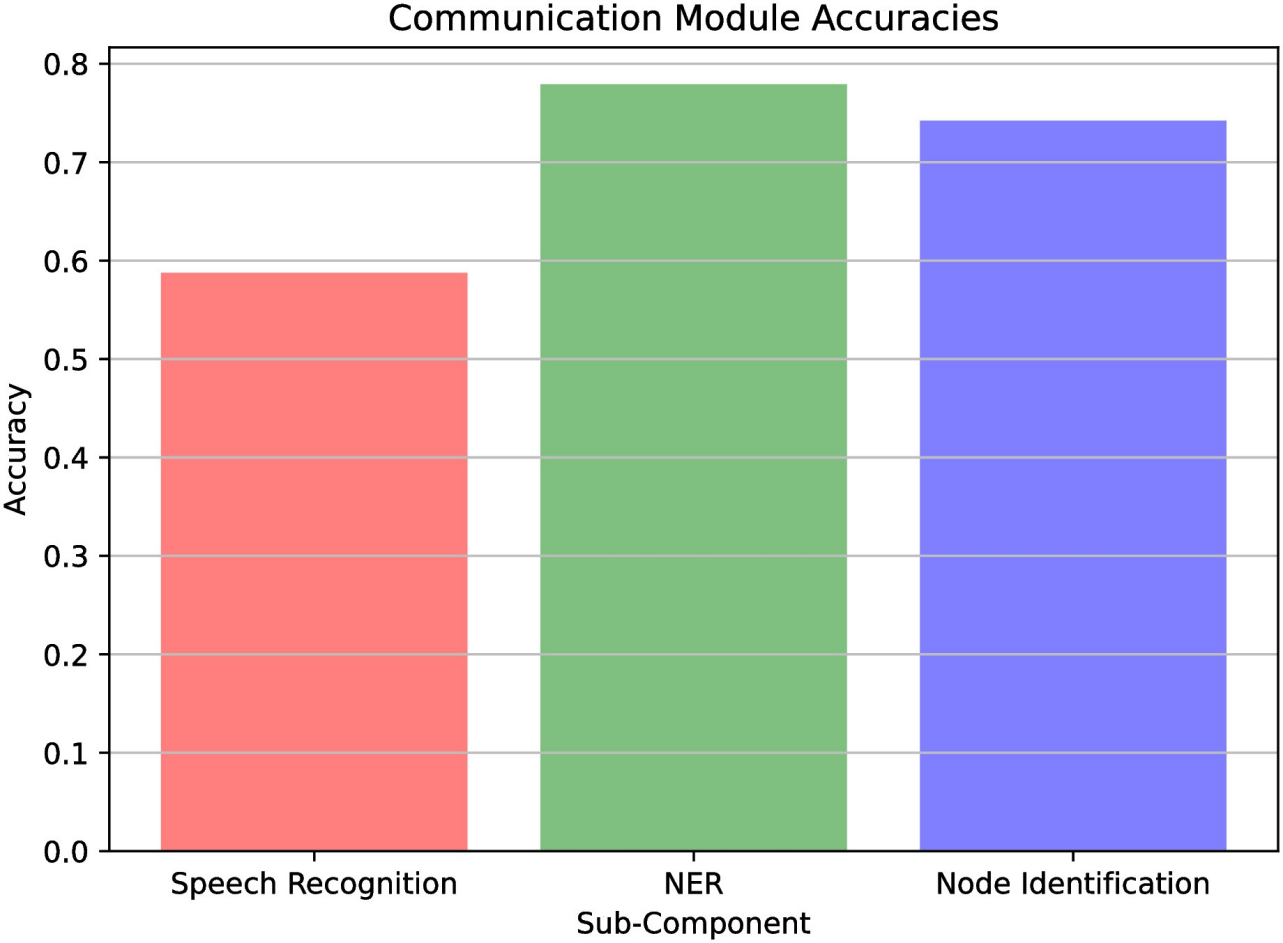
NLP modules accuracies. The accuracies for the three NLP modules—Speech Recognition (58.62%), NER (77.77%), and Node Identification (74.07%).

We also break down the evaluation of the NLP modules into three parts: i) the speech recognition module, ii) the NER module, and iii) the node identification module. Then, having recorded all the human’s spoken phrases plus the outputs of the three modules, in all time steps of the trials, we start reporting the accuracy of the speech recognition module. We also recorded, for each sentence, whether or not the sentence contained the information necessary to identify the human’s location. Finally, from this set of informative phrases, we evaluate the accuracy of the NER module and, subsequently, of the node identification module. The values depicted in Fig 10, which plots the accuracies for these three modules in a live environment, average over all iterations of the seven experiment participants.

From Fig 10, we observe that perfectly recognizing human speech is the hardest task of the three, with only a performance of 58.62%. From the spoken phrases that are informative enough to infer the correct human location, NER was able to identify the correct location in 77.77% of them. At last, the node identification module, which takes as input the NER location string, was able to correctly output the right human nodes 74.07% of the time.

### Additional studies

In this section, we present two additional experiments to assess both the impact of information sharing on team performance and the scalability of our decision approach, TEBOPA, to larger domains.

#### Disabling communication

To further evaluate the impact of communication on team performance, we conducted an additional experiment where we removed the possibility of communication between the robot and the human. The same modeling described in The Decision Module section holds, with the exception that there is now no *locate human* action available to the ad hoc robot. The same previous tasks are kept as well as the human model.

Given that the location of the human, when queried, is given to the ad hoc robot as part of its observation, this ablation can be seen as a setting where the available information to the robot is reduced. Given that our approach deals with the task identification problem of ad hoc teamwork by using any information available, we expect that the less information we have, the better. To test this hypothesis, we evaluate a total of four approaches:

- TEBOPA: Our original ad hoc approach which doesn’t know the correct task beforehand and is able to query the human for its location;
- Optimal policy under partial observability: A non-ad hoc ablation of our approach which knows the correct task beforehand and is able to query the human for its location;
- TEBOPA without communication: Our original ad hoc approach which doesn’t know the correct task beforehand and doesn’t have the action for locating the human within its action space;
- Optimal policy under partial observability without communication: A non-ad hoc ablation of our approach which knows the correct task beforehand and doesn’t have the action for locating the human within its action space.

**Fig 11:**
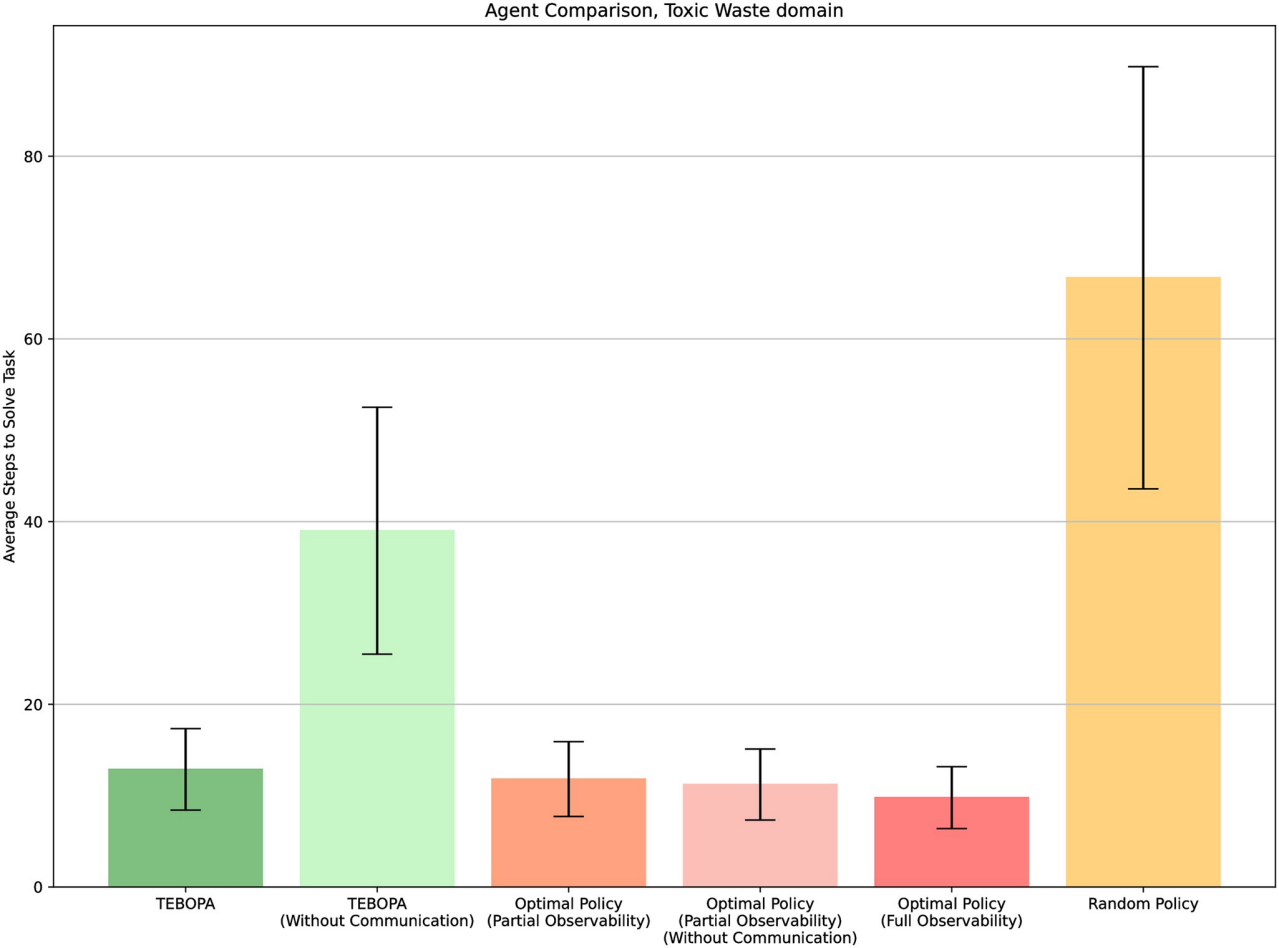
Disabled communication experiment. Average steps to complete a task for different approaches in the disabled communication experiment. Error bars correspond to a confidence interval with confidence of 95% (*α* = 0.05), calculated over a total of 32 independent trials.

All agents are evaluated for 32 independent trials. We conduct this experiment in the simulated environment, given its shown reproducibility of real-world results in our main evaluation section. Fig 11 displays the average steps to solve the tasks over the 32 independent trials. We keep as a reference, the optimal policy (which has full observability of the environment) and random policy, for best and worst possible performances, respectively.

From these results, two main observations can be made. First, when the task is known, following the optimal policy (under partial observability) showcases no statistical difference when communication is disabled (*α* = 0.05). This result is expected, since going directly to the nodes containing the waste and waiting for the human is an optimal strategy if the location of the balls is known beforehand, rendering the problem as a problem of self-location instead of coordination with another teammate. The second and most important observation we can make from these results is that when we’re dealing with an unknown task in an ad hoc setting, removing the possibility of querying the human for its location yields a significant drop in performance, as shown by the results obtained by our approach, TEBOPA, and our approach without communication (*p* = 0.000656, *α* = 0.05). The most plausible explanation for this observation is that the availability of the human’s location in each time step allows the ad hoc robot to substantially enhance its belief in the correctness of the task being executed. Without communication, the robot is only able to observe where it is and when a ball is placed within its compartment, which happens at most three times throughout the episode and may not be enough information to immediately identify the correct task. These results show that without communication, the problem of ad hoc teamwork under partial observability becomes even harder.

#### Scaling to larger problems

We conclude our evaluation by conducting an additional experiment to assess the scalability of our decision module. The Toxic Waste domain has environments with a total of |X|=260 possible states, |Z|=36 possible observations, and $|A_{0}|=5$ actions. Given that these spaces are relatively small, we conduct an additional experiment in the Predator-Prey (or Pursuit) domain [36], a benchmark from the multi-agent systems community where a team of predator agents has the goal of capturing a moving prey. This domain is relatively larger for a team of two agents, with environments now with a total of |X|=626 states, |Z|=81 possible observations, and $|A_{0}|=6$ actions.

**Fig 12:**
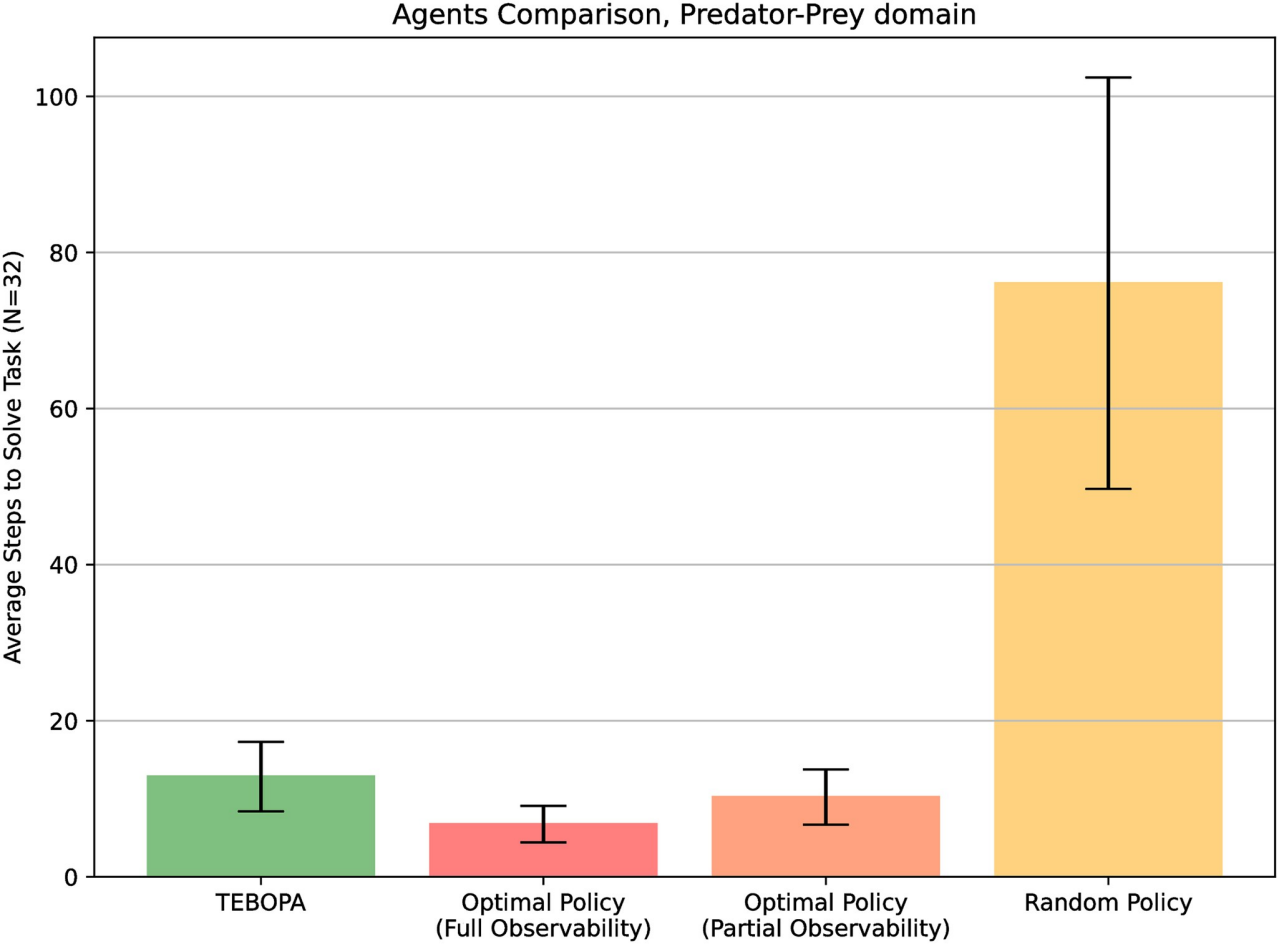
Predator-Prey domain results. Average number of steps to complete a task in the Predator-Prey domain. Error bars correspond to a confidence interval confidence of 95% (*α* = 0.05), calculated over all trials.

Similar to the Toxic Waste domain, we compare, in the Predator-Prey domain, our approach against (i) an optimal agent, which has full observability of the environment, (ii) an approach that knows the correct task and acts optimally under partial observability, and (iii) a random baseline. All approaches are evaluated in *N* = 32 independent trials. Fig 12 showcases the average steps to complete the tasks over the 32 trials. Tasks are selected randomly in each trial, and in the predator-prey domain, corresponding to the direction from which each predator must surround the prey for the team to capture it.

As expected, results show the optimal agent to have completed, on average, trials in 6.75 steps, solving episodes in fewer steps than the optimal agent under partial observability (*p* = 0.007, *α* = 0.05), TEBOPA (*p* = 0.00012, *α* = 0.05) and the random baseline (*p* = 0.00000001, *α* = 0.05). The results for the optimal agent under partial observability and our approach, TEBOPA, show no statistical difference for *p* > *α* = 0.05, showcasing similar performances by solving episodes in an average of 10.22 and 12.84 steps, respectively. They both solve episodes in fewer steps than the random baseline (*p* = 0.00000004, *α* = 0.05 for the optimal agent under partial observability and *p* = 0.000000088, *α* = 0.05 for TEBOPA). Finally, as expected, the random baseline took significantly longer steps to solve episodes, with an average of 76.06 steps. These results allow us to conclude that even in a significantly larger domain, our approach is able to correctly identify the correct task being performed by a teammate and solve it in a near-optimal number of steps.

## Conclusions and future work

Ad hoc teamwork addresses the decision-making problem of an agent when teamed to work with other unknown agents. Without any prior coordination or communication protocol, the agent must infer the cooperative task being performed, identify the teammates, and act to complete the task effectively. This demands the agents to adapt and engage in dynamic problem-solving without prior knowledge or extensive planning.

In this work, we present HOTSPOT, a novel framework for ad hoc teamwork in human-robot teams. Specifically, our framework has two main modules, addressing the two key challenges in the interaction between a robot and a human teammate within ad hoc teamwork scenarios. The first module handles all the task-related decision-making challenges (i.e., task identification, teammate identification, and planning), and is responsible for orchestrating the robot’s contributions to the collaborative effort. The second module deals with the communication challenge between robots and humans by employing NLP techniques, which enables the exchange of information between the robot and the human being, therefore enhancing the overall efficiency of the collaborative effort.

To evaluate our framework, we use a task that involves a mobile robot and a human teammate in a cooperative task of collecting objects in an open space, illustrating the main features of our framework in a real-world task. The Results section shows that our approach always identified and completed the task with a near-optimal number of steps while using partial and imperfect information. We also observe that, on average, the proposed approach identified the task faster than the optimal policy, showing the potential that this approach has in a real task environment and highlighting the practicality and robustness of our framework in addressing the challenges of ad hoc teamwork.

Although our approach has shown excellent results, we can always incorporate enhancements to further improve the proposed methods’ performance.

The main limitation of our work is its evaluation in a turn-based scenario, where the human and the robot act in turns. The next logical line of work would be to extend our approach to real-time tasks, where both the human and the robot are free to move when they want. In this sense, the exploration of real-time collaborative scenarios presents a promising avenue for future research and development, and adapting our framework to such dynamic environments will require the integration of responsive and adaptive decision-making processes that can handle the complexities of simultaneous human and robot actions. This aligns with the evolving landscape of human-robot interaction and will extend the applicability of the approach to real-world scenarios.

Another limitation is the speech recognizer, which currently uses an online recognition service, that is not customized for the restricted vocabulary used in human-robot conversation and is not suitable for the noisy environment of human-robot interactions. Moreover, besides the low average performance, any instability in the robot’s Internet connection makes its use unfeasible. In this sense, developing an offline system customized for our domain would bring enormous advantages, both for the classifier performance and the response speed of the decision module.

Another possibility for future work is to explore domains with arbitrarily large continuous state spaces, for which tabular methods like the ones used do not scale well. In such domains, function approximation methods can be employed, such as feed-forward neural networks combined with long short-term attention cells or attention-based mechanisms to extract latent states directly from observations.

Also, we plan to invest in other types of sensors for the robot, especially those that capture 360-degree scenes. This will empower the robot to gain a comprehensive view of its surroundings, enabling more robust and context-aware decision-making processes. That way, independent of the robot’s position and orientation, it will be possible to apply computer vision techniques to enhance the robot’s observation of the environment.

## Methods

### Ethics statement

Human Subject Research (involving human participants and/or tissue)

- Name of the ethics committee that approved the study: Ethics Committee of Instituto Superior Técnico (EC-IST)
- Approval number: 20/2019 (EC-IST)
- Form of consent obtained: Oral, after the invitation to participate in the study and before conducting the trials. If an individual did not consent, they would not participate in the study (no cases).
